# Variation of the photoluminescence spectrum of InAs/GaAs heterostructures grown by ion-beam deposition

**DOI:** 10.3762/bjnano.9.261

**Published:** 2018-11-02

**Authors:** Alexander S Pashchenko, Leonid S Lunin, Eleonora M Danilina, Sergei N Chebotarev

**Affiliations:** 1 Laboratory of Nanotechnology and Solar Energy, Federal Research Center Southern Scientific Center of Russian Academy of Sciences, 344006, 41, Chekhov Avenue, Rostov-on-Don, Russia; 2 Department of Physics and Electronics, Platov South-Russian State Polytechnic University (NPI), 346428, 132, Prosveshchenia str., Novocherkassk, Russia

**Keywords:** infrared photodetectors, ion-beam deposition, nanoheterostructures, photoluminescence, quantum dot, semiconductors

## Abstract

This work reports on an experimental investigation of the influence of vertical stacking of quantum dots, the thickness of GaAs potential barriers, and their isovalent doping with bismuth on the photoluminescence properties of InAs/GaAs heterostructures. The experimental samples were grown by ion-beam deposition. We showed that using three vertically stacked layers of InAs quantum dots separated by thin GaAs barrier layers was accompanied by a red-shift of the photoluminescence peak of InAs/GaAs heterostructures. An increase in the thickness of the GaAs barrier layers was accompanied by a blue shift of the photoluminescence peak. The effect of isovalent Bi doping of the GaAs barrier layers on the structural and optical properties of the InAs/GaAs heterostructures was investigated. It was found that the Bi content up to 4.96 atom % in GaAs decreases the density of InAs quantum dots from 1.53 × 10^10^ to 0.93 × 10^10^ cm^−2^. In addition, the average lateral size of the InAs quantum dots increased from 14 to 20 nm, due to an increase in the surface diffusion of In. It is shown that isovalent doping of GaAs potential barriers by bismuth was accompanied by a red-shift of the photoluminescence peak of InAs quantum dots of 121 meV.

## Introduction

The formation of III–V nanoheterostructures with quantum dots (QDs) raises the possibility of developing a new generation of photodetectors in the infrared range [1–3]. The significant problems of existing HgCdTe detectors are low yield and high cost in comparison with quantum-well infrared photodetectors (QWIPs) [4]. The QWIPs, in turn, have a simpler technology but a low quantum efficiency and require cooling. One way to solve these problems is to grow semiconductor heterostructures in which QDs are embedded. The localization of photogenerated charge carriers in a quantum dot along three directions leads to a low thermionic emission and decreases the dark current [5–6]. InAs/GaAs, Ge/Si, and others are examples of such heterostructures [7–14]. The structural and optical properties of InAs QDs rely heavily on their surrounding matrix, which is separated into two parts: the top with a GaAs strain-reducing layer; the bottom with a strained buffer layer (wetting layer) [15–19]. Thus, the simplified energy band diagram of the active region of an InAs/GaAs heterostructure is an InAs quantum dot built into a GaAs matrix in the form of a quantum well. It follows from the above that it is possible to manipulate the photosensitivity spectrum of heterostructures through three methods: 1) the size, shape and density of the quantum dot; 2) the vertical stacking of quantum dot arrays; 3) the material of the barrier layers surrounding the quantum dots. We and other researchers described the first method in [20–23]. Mechanical strains are a technological problem in the development of effective IR devices based on InAs/GaAs nanoheterostructures with vertically stacked QD layers [24–25]. The surface density and geometric sizes of InAs quantum dots varies depending on the mechanism and the sign of the stresses, and consequently, the photoluminescence properties of InAs/GaAs heterostructures will vary too. The distinctive feature of the first and second methods is that the optical transitions are performed inside the potential well formed by the quantum dot. In the case of using the third method, other mechanisms of optical transitions are possible.

The growth of QDs occurs in the Stranski–Krastanov mode, when the substrate wetting layer is elastically stressed. An InAs/GaAs heteropair is ideal for creating conditions for a transition from 2D to 3D growth but has some limitations for a number of functional applications such as manipulating the photosensitivity spectrum in the infrared region. The solution to the problem can be found by the selection of a potential-barrier material for QDs based on III–V multicomponent solid solutions. GaAsSb [26], InGaAs [27], InGaAsN [28–29] and GaAsNSb [30–31] have been used for these purposes. In this work, it is proposed to use GaAsBi. Bismuth is an isovalent doping and forms ternary, quaternary and quinary solid solutions with III–V compounds [18,32–38]. Bi incorporation into GaAs makes it possible to change the lattice mismatches. The GaAs energy band diagram varies greatly due to the large size of Bi atoms. Emerging strains in the GaAsBi layer should have an influence on the surface density and sizes of InAs QDs and also on the photoluminescence properties of InAs/GaAs heterostructures [17–18,20,32–33]. In this regard, a study of the influence of Bi on the optical properties of InAs/GaAs heterostructures is an actual requirement.

In this work, ion-beam deposition (IBD) is used to grow the samples. IBD has significant advantages over many methods of growth from the gas phase, since it allows for a control of the energy of sputtered atomic fluxes and their interaction with the growth surface [39]. IBD plays an important role in the semiconductor materials technology [40–48].

In this work, we study the influence of QD vertical stacking, the thickness of GaAs potential barriers and their doping by bismuth on the structural and photoluminescence properties of InAs/GaAs heterostructures.

## Experimental

### Heterostructures with vertically stacked InAs QDs

Several samples were grown to investigate the influence of QD vertical stacking on the photoluminescence spectrum of the heterostructures. The heterostructures were synthesized by using an IBD system [21]. The deposition experiments were performed on GaAs(100) substrates. The deposition of the material was carried out by sputtering the target with an Ar^+^ ion beam. Calibration dependencies of InAs and GaAs sputtering yields on the beam energy, the slope angle and the flux density were obtained earlier [21,49–50]. Two types of samples were grown for the research of vertically stacked QDs. The first type of samples (ST#1) contained one to three QD layers separated by 15 nm GaAs layers. The second type of samples (ST#2) contained three QD layers that were separated by GaAs barriers of 15, 20 and 30 nm.

During the growth process, pressure in the vacuum chamber was 3.7 × 10^−7^ Pa. The 500 nm thick n^+^-GaAs buffer layer at *T* = 610 °C was grown first. The accelerating voltage of the ion beam was 450 V, and the current density was 3.2 × 10^−4^ A/cm^2^. Furthermore, the i-GaAs barrier layer was deposited under similar conditions, then a 15 second pause was made, and the temperature was reduced to 535 °C. InAs QDs were grown at an accelerating ion voltage of 250 V and an ion current density of 4.5 × 10^−6^ A/cm^2^. The covering of QDs by the i-GaAs layer was accompanied by an increase of the temperature to 550 °C.

### InAs/GaAs heterostructures with potential barriers of GaAsBi

Growth of GaAs_1−_*_x_*Bi*_x_* layers was carried out using polycrystalline targets, which were made as follows. The batch composition was calculated in accordance with the solid phase composition of the finished GaAs_1−_*_x_*Bi*_x_* target. The total mass of the melt was calculated taking into account the ingot volume of the polycrystalline target. After chemical treatment, the components of the batch were placed in a graphite container. Heating of the container with the batch was carried out by a resistive heater to a temperature above the melting point of GaAs (1250 °C), which is the highest-melting compound. The melt was kept for 10 min under such conditions, then rapid shutdown of the heating elements was followed by crystallization of the initial melt. The resulting polycrystalline ingot was cut to 2 mm thick plates.

For investigation of the influence of the potential barrier material on the photoluminescence properties of InAs/GaAs heterostructures, experimental samples with GaAs_1−_*_x_*Bi*_x_* potential barriers with bismuth fractions of 1, 3 and 5 atom % were grown. One experimental sample contained GaAs barriers for comparison. The Bi-containing layers were grown at 360 °C. The samples contained one layer of InAs QDs.

### Characterization methods

Photoluminescence (PL) measurements of InAs/GaAs heterostructures were carried out at temperatures of 90 and 300 K in the spectral range from 0.9 to 1.4 eV. An injection laser with 402 nm wavelength and power of 12.5 mW was used as a source of excitation optical radiation. The PL signal was recorded by an MDR-23 monochromator with a cooled germanium photodiode PDG-3600. Protection of the monochromator entrance slit from the reflected harmonics of the exciting laser radiation was carried out by means a Y-1.4 optical filter (light yellow color).

A study of the surface morphology of the grown heterostructures was carried out using a Solver HV atomic force microscope. The structural properties of the GaAsBi/GaAs, InAs/GaAs, and InAs/GaAsBi heterointerfaces were investigated by methods of Raman spectroscopy and X-ray diffraction (XRD). X-ray diffraction reflection curves were investigated on a high-resolution TRS-1 X-ray diffractometer with a third-crystal geometry using the Cu Kα emission line (λ = 0.154 nm). A Renishaw InVia Raman spectrometer was used for Raman investigations.

## Results and Discussion

### Photoluminescence properties of InAs/GaAs heterointerfaces

The photoluminescence properties of vertically stacked QD arrays grown by using molecular beam epitaxy (MBE) are well studied in [24–25]. But the IBD method differs from MBE. Therefore, it remains relevant to research the vertical stacking of QDs grown by using IBD. In the beginning, ST#1 samples were studied. The statistical analysis by the threshold tool in the Image Analysis 2.1.2 program showed that the density of QDs in the bottom layer reached 1.42 × 10^10^ cm^−2^, the average lateral size of the quantum dots was 17 nm, and their height was 6.3 nm.

**Figure 1:**
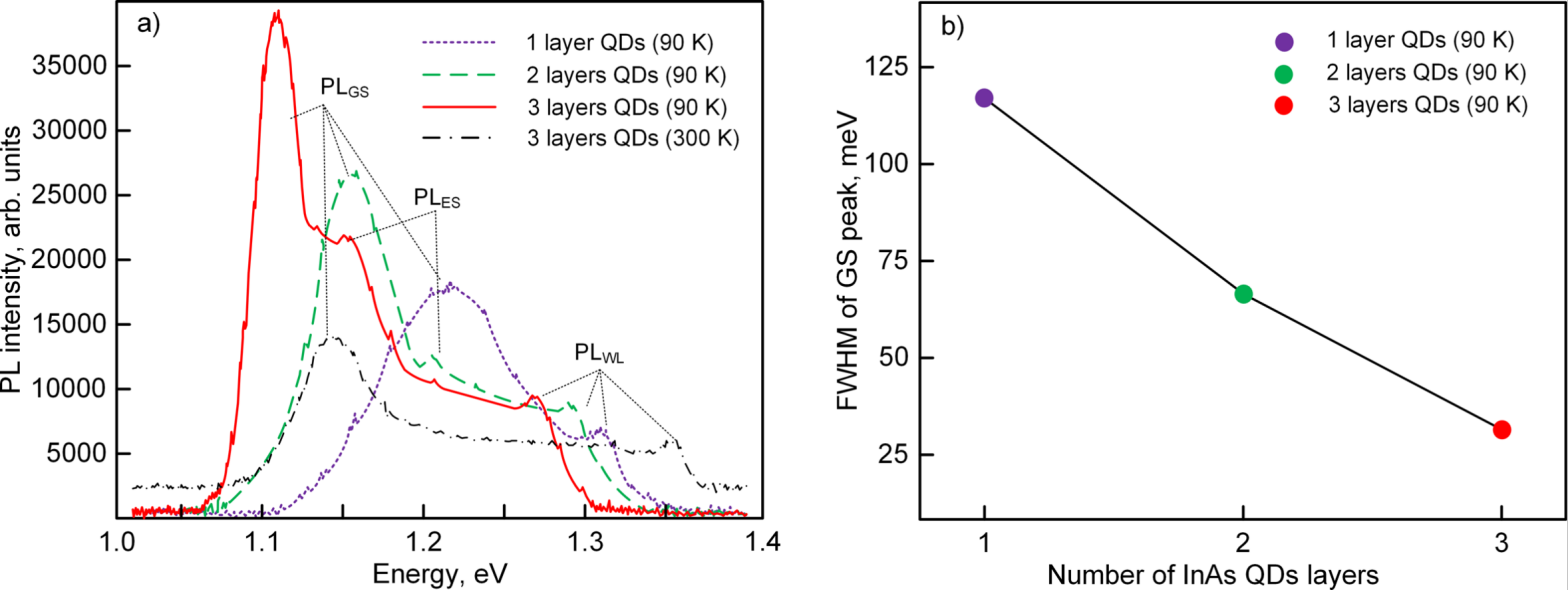
a) Photoluminescence spectra of samples with vertically stacked InAs QD arrays; b) full width at half maximum of ground state peaks as a function of the number of InAs QD layers.

During PL excitation, certain steady-state conditions are established in the sample, in which the radiation rate depends on the lifetime of the photogenerated charge carriers in the QD or in the wetting layer. Therefore, the photoluminescence in the grown samples can go through the ground (PL_GS_) and excited (PL_ES_) states of the electrons at the QDs and also through the energy levels in the wetting layer (PL_WL_). The measured PL spectra of the vertically stacked QD arrays are shown in Figure 1.

The appearance of PL_WL_ peaks indicates the Stranski–Krastanov growth mode of QDs. The photoluminescence of a sample with one QD layer is characterized by the absence of a peak of the excited states, and the PL_GS_ peak has a full width at half maximum (FWHM) of ca. 118 meV with a maximum at an energy of 1.22 eV (Figure 1a). The spectra of samples with two and three QD layers differ by a decrease of FWHM of the PL_GS_ peaks (Figure 1b) and also by an appearance of PL_ES_ peaks. In this case, the PL_GS_ peaks in the samples with QD vertical stacking are shifted to lower energies (red-shift). The shift value between PL_WL_ peaks and PL_GS_ peaks in the samples increases by 70 meV. It is seen that the FWHM of PL_GS_ peaks decreases with increasing number of InAs QD layers (Figure 1b). As a result, the luminescence spectrum of vertically stacked QD arrays narrows and becomes similar to the large single-QD spectrum [51–53].

The red-shift is also described in [24] for heterostructures with five QD layers of InAs and in [25] for heterostructures with 1–20 QD layers of In_0.5_Ga_0.5_As. In both cases, the heterostructures were grown by using MBE. As a comparison of the possibilities of methods for the formation of heterostructures, we note that the average lateral sizes of quantum dots are the same, and their height, in the case of IBD, is higher. The reasons for this may be related to the difference in the formation of growth fluxes. In IBD, much depends on the calibration of the sputtering yield of the target, which affects the kinetics of the surface processes on the substrate.

**Figure 2:**
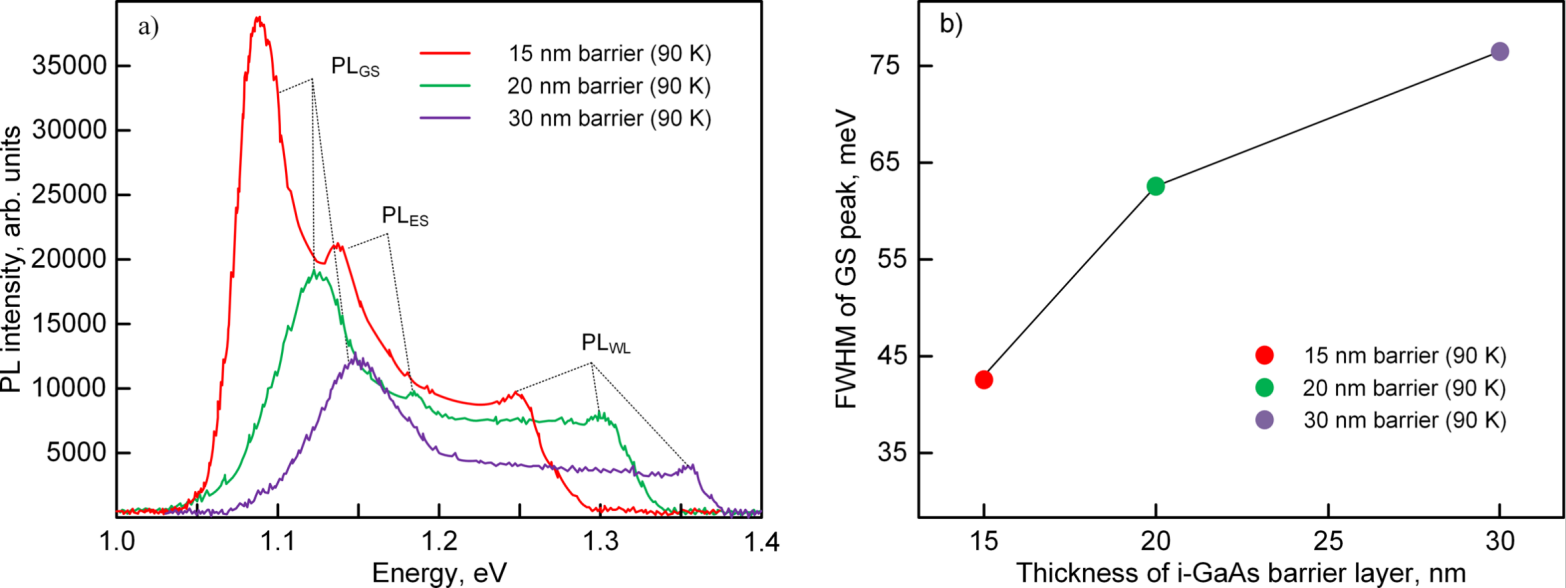
a) Photoluminescence spectra of the InAs/GaAs heterostructures with different thicknesses of the GaAs barrier layer; b) full width at half maximum of ground state photoluminescence peaks as a function of the thickness of the i-GaAs barrier layer.

The influence of the thickness of the i-GaAs barrier layer (ST#2 samples) on the photoluminescence properties of the InAs/GaAs heterostructures is shown in Figure 2. It is seen that a decrease in the i-GaAs layer thickness from 30 to 15 nm is accompanied by a red-shift of the PL peaks with an increase in the intensity (Figure 2a) and decrease of FWHM (Figure 2b).

The energy difference between PL_GS_ and PL_WL_ peaks decreased from 205 to 158 meV when the barrier layer thickness was changed from 30 to 15 nm. Obviously, the thicker the i-GaAs layer, the less it is strained. The coating of QDs with a thin i-GaAs layer creates the conditions for the inheritance of properties from the bottom QD layer by a new layer of InAs QDs. Otherwise, if the thickness of the i-GaAs layer is much higher than the height of the QDs, their sizes and density will be determined by the self-organization on the new growth surface, which can cause a fluctuation in QD sizes during vertical stacking and a degradation of the structural and optical properties of the InAs/GaAs heterostructures. The results of the investigations showed that in the case of three QD layers of InAs separated by 15 nm i-GaAs barriers, the density of the array decreased insignificantly from 1.42 × 10^10^ to 1.33 × 10^10^ cm^−2^. In addition, the average lateral size of the QDs increased by 2 nm. For 30 nm layers of i-GaAs, the average lateral size of the QDs is increased by 2.6 nm, while the density of the top array of quantum dots is decreased to 1.27 × 10^10^ cm^−2^. A similar situation was observed in [24,54]. A significant increase in the characteristic QD sizes during vertical stacking was described in [25] only after the deposition of ten QD layers through thin barriers. The observed effect of red-shift and simultaneous increase of PL intensity with increasing number of QD layers was also described in [24] and mainly is explained by a suppression of relative oscillations of QD heights accompanied by high homogeneity of lateral QD sizes. Thus, a decrease in the i-GaAs layer thickness, on the one hand, limits the fluctuation of QD heights in vertically stacked arrays and stabilizes them and, on the other hand, improves the charge transfer due to the resonant tunneling between the vertical InAs QD layers.

### Photoluminescence properties of InAs/GaAsBi heterointerfaces

**Figure 3:**
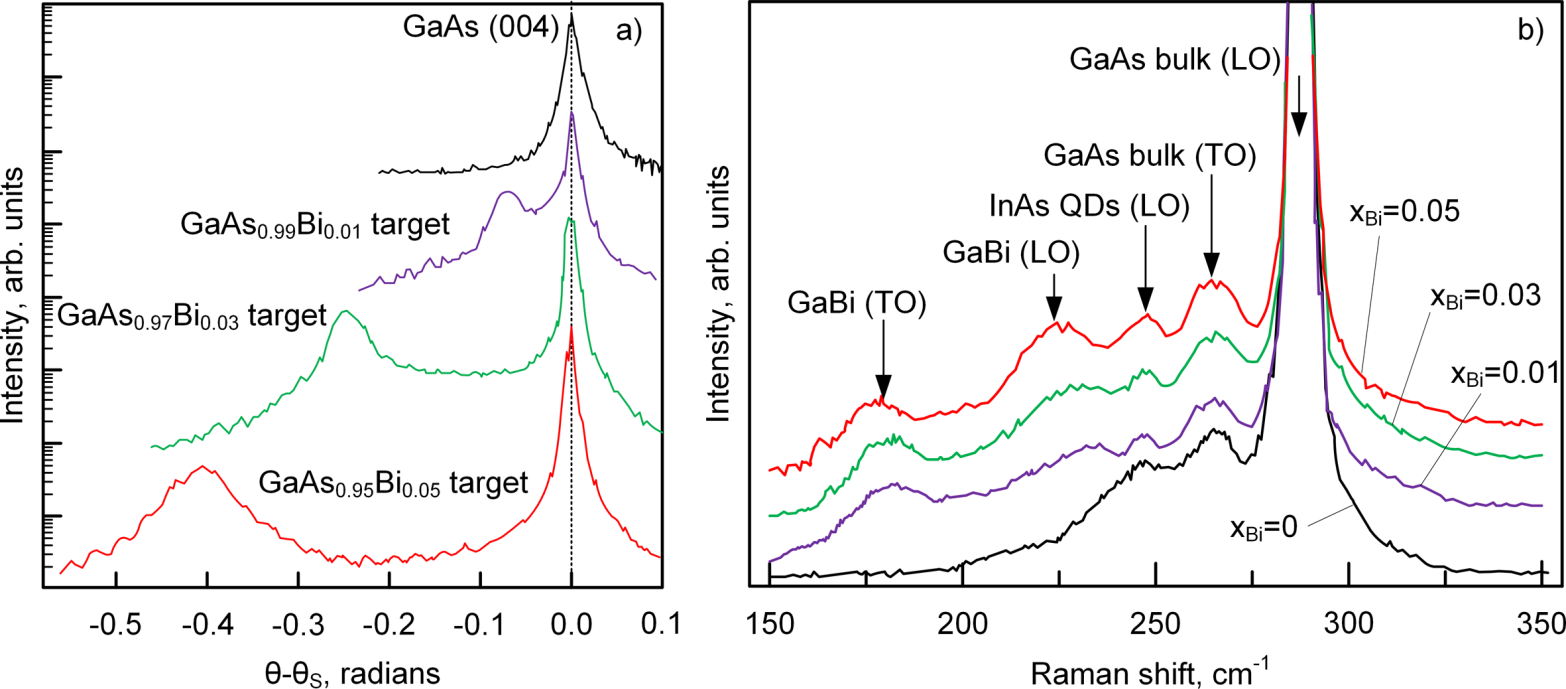
a) XRD spectra of GaAs_1−_*_x_*Bi*_x_* films grown on GaAs(100) substrates; b) Raman spectra of InAs/GaAs and InAs/GaAs_1−_*_x_*Bi*_x_* heterostructures.

To study the photoluminescence properties of InAs/GaAsBi heterointerfaces, it was necessary to investigate the morphology and structural properties. At first it was necessary to verify the conservation of the stoichiometric composition of GaAs_1−_*_x_*Bi*_x_* during ion-beam deposition. For this purpose, GaAs_1−_*_x_*Bi*_x_* films were grown on GaAs(100) substrates. The thickness of the GaAs_1−_*_x_*Bi*_x_* layers was 20 nm. The results of XRD studies of the epitaxial layer of GaAsBi are shown in Figure 3a.

Bi is an isovalent dopant material for III–V compounds and replaces group-V element. Consequently, the GaAs_1−_*_x_*Bi*_x_* crystal lattice should be a zinc blende lattice. Therefore, a diffraction maximum corresponding to the GaAs(004) lattice planes was measured. This is due to its high structure factor, which governs the maximum diffraction intensity. In Figure 3a, two main peaks are identified: a sharp peak of high intensity at 0 radians (θ_s_ = 33°) from the GaAs substrate, and low-intensity peaks with negative Δθ due to the strained GaAs_1−_*_x_*Bi*_x_* epitaxial lattice. That kind of peak splitting (with respect to the substrate) is usually observed in epitaxial layers with lattice relaxation. The Bi contents were calculated from obtained X-ray diffraction patterns over the linear dependence *x* = 6.77Δθ under the condition of a completely strained GaAs_1−_*_x_*Bi*_x_* layer [55]. The calculated values of bismuth contents were *x*_Bi_ = 0.99%, 2.98% and 4.96%. The composition of GaAs_1−_*_x_*Bi*_x_* epitaxial layers differs from the composition of the sputtering targets. In our opinion, this difference is due to the desorption of Bi from the surface, despite the low deposition temperature of 360 °C. The influence of bismuth on the structural properties of InAs/GaAsBi and InAs/GaAs heterointerfaces was investigated by using Raman spectroscopy.

In accordance with the selection rule, modes of longitudinal optical phonons (LO) and longitudinal acoustic phonons (LA) are allowed in the heterointerfaces. The Raman spectroscopy results are shown in Figure 3b. In addition to the allowed modes, forbidden transverse optical (TO) modes for optical phonons of bulk-like GaAs (268 cm^−1^) and GaBi (182 cm^−1^) are also observed. This indicates a change in the selection rule under Raman scattering in the InAs/GaAs system caused by lattice strain during the incorporation of Bi into the GaAs matrix. In the spectra we can clearly see the longitudinal modes of optical phonons of bulk GaAs (LO) at 283 cm^−1^, InAs QDs (LO) at 248 cm^−1^ and GaBi at 235 cm^−1^. Effects not detectable in the Raman spectrum of the InAs/GaAs heterostructure appear in the spectra of InAs/GaAs_1−_*_x_*Bi*_x_* samples in the interval of 150–250 cm^−1^. An increase in *x*_Bi_ is accompanied by the Raman shift of the GaAs-like and GaBi-like peaks to smaller wavenumbers. Due to decrease of lattice mismatch and, thus, less strain in the InAs/GaAs_1−_*_x_*Bi*_x_* heterointerface compared to InAs/GaAs, there is a Raman shift of the peaks of InAs/GaAs_1−_*_x_*Bi*_x_* heterostructures relative to the spectrum of the InAs/GaAs heterostructure. The results of Raman spectroscopy are in good agreement with XRD.

**Figure 4:**
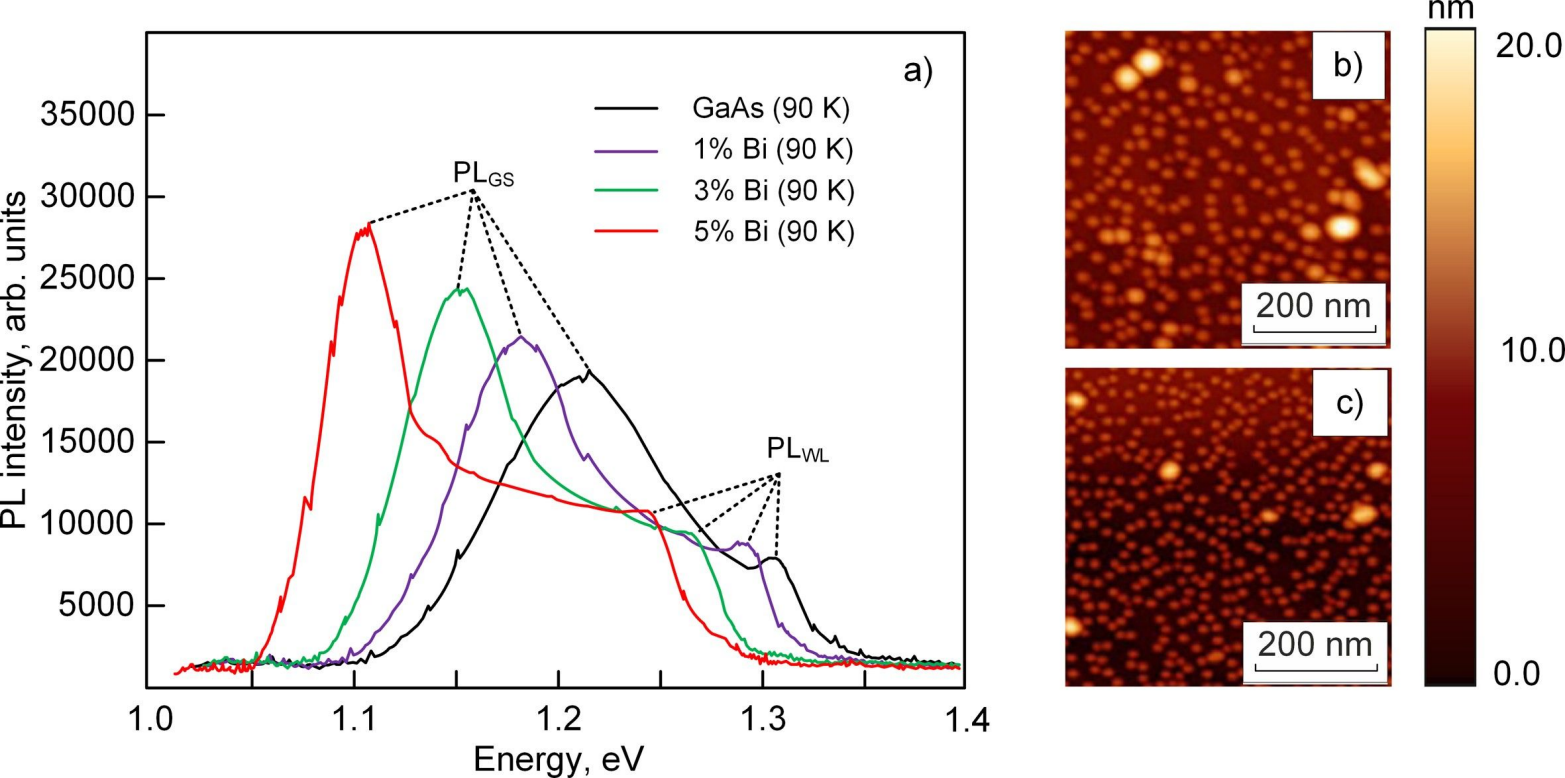
a) Photoluminescence spectra of InAs/GaAs heterostructures with different Bi content; b) Atomic force microscopy images of the morphology of InAs QDs on GaAs_0.95_Bi_0.05_; c) Atomic force microscopy images of the morphology of InAs QDs on GaAs.

Features of PL in the InAs/GaAs_1−_*_x_*Bi*_x_* heterostructures are shown in Figure 4a.

**Figure 5:**
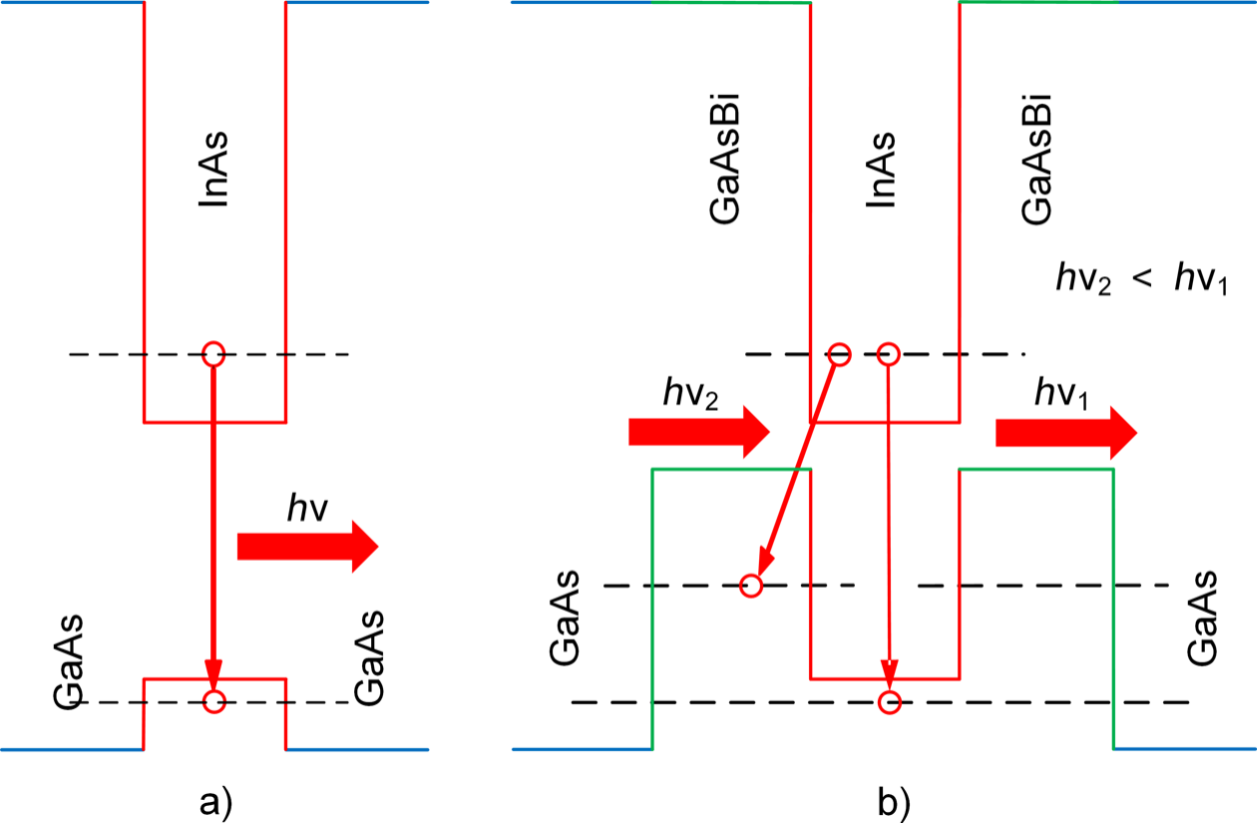
Energy band diagram of heterostructures: a) InAs/GaAs; b) InAs/GaAs_1−_*_x_*Bi*_x_*.

The difference between the energies of the PL_GS_ peaks of a sample without Bi and a sample with *x*_Bi_ = 4.96 atom % is 121 meV. This result differs from the red-shift of 23 meV obtained in [56] at room temperature. The reason for this is that InAs QDs in our work have larger sizes and lower density. The results of the investigation of the surface morphology with InAs QDs in samples with GaAs and GaAs_1−_*_x_*Bi*_x_* potential barriers are shown in Figure 4b,c. It is seen that there is a decrease of InAs QD density at growth on the GaAs_0.95_Bi_0.05_ surface. The AFM analysis showed that the density of QDs in InAs/GaAs_0.95_Bi_0.05_ and InAs/GaAs heterosystems was 0.91 × 10^10^ cm^−2^ and 1.53 × 10^10^ cm^−2^, respectively. Doping GaAs with bismuth is accompanied by an increase in the average size of QDs from 14 to 20 nm. The results can be explained by an increasing rate of In surface diffusion on GaAs_1−_*_x_*Bi*_x_* during the ion-beam deposition due to the Bi surfactant effect. Note that scientists have different points of view about it. Some argue that doping of Bi during the QD growth process by MBE [17–18,20] and metal-organic vapor-phase epitaxy (MOVPE) [32–33] reduces the In adatom diffusion length and prevents the coalescence of InAs QDs. Then, the QD array density increases. Others argue that the usage of Bi during MOVPE and MBE of InAs QDs [57–58] enhances In adatom diffusion length. In this case, QD sizes increase, and the QD density decreases. Detailed analysis of these works elucidated that in the first case, doping with bismuth was being done during InAs QD growth on GaAs. In the second case [57–58], the QD growth was being done on GaAs_1−_*_x_*Bi*_x_* surfaces. Among other things, temperature conditions also differed from each other. In [20,32–33], doping with Bi on InAs QDs was carried out at 510–530 °C. In our case, as in [52–53], GaAs_1−_*_x_*Bi*_x_* layers were grown at temperatures below 500 °C. We also want to emphasize the fact that the covalent radii [59] of Bi = 148 pm and In = 142 pm atoms are very close. Therefore, in the InAs QD growth process on the GaAs_1−_*_x_*Bi*_x_* surface, In adatom surface diffusion can be realized both through substituting Ga or Bi vacancies. Increase of QD heights in the InAs/GaAs_0.95_Bi_0.05_ heterosystem is an indirect demonstration of this. Bi has a large atomic size, so it creates elastic strain in the GaAs lattice and thus influences the QD self-organization. Consequently, using Bi as a surfactant on the GaAs growth surface changes the kinetics of In surface diffusion during ion-beam deposition. Unchanged is the circumstance that with an increase in Bi content in GaAs, an increase in the intensity of photoluminescence is observed in spite of a decrease in InAs QD density (Figure 4b,c). The effect is due to the fact that Bi incorporation leads to valence-band splitting of GaAs, shifting the energy maximum of heavy holes deep into the energy gap. This induces a change in the profiles of the valence and the conduction bands of the InAs/GaAs heterostructure. The InAs/GaAs_1−_*_x_*Bi*_x_* heterointerface forms a type-II misaligned heterojunction [60]. In accordance with the band diagram in Figure 5, the profile of the valence band of the GaAs_1−_*_x_*Bi*_x_* layer shifts in such a way that it forms a well for holes on either side of the InAs QD. In this case, the optical transitions in the direction “quantum dot–barrier layer” begin to appear. The energy of optical transitions is less than the energy of transitions through the ground states of QDs, which explains the red-shift of the PL peaks (Figure 5b). In addition, we suppose that an increase in the lateral QD sizes also contributes to the red-shift of the photoluminescence peak.

Note that in this case density reduction of the InAs QDs is not a negative factor. QDs being at a more remote distance from each other are able to form a narrower energy sub-band in the conduction band. This effect can be used for the fabrication of multicolor photodetectors.

## Conclusion

The properties of InAs/GaAs and InAs/GaAsBi heterointerfaces grown by ion-beam deposition are analyzed. Methods of controlling the photoluminescence spectrum of InAs/GaAs heterostructures are considered. It is demonstrated that the vertical stacking of three QD layers decreases the FWHM of the photoluminescence peaks. According to the studies of photoluminescence, it is established that the variation of the photoluminescence spectrum of InAs/GaAs heterostructures can be carried out by isovalent doping of potential barriers for QDs. It is shown that a content of 4.96 atom % Bi in GaAs leads to a red-shift of the ground-state peaks of the QD array of 121 meV and a decrease of the FWHM. The XRD and Raman spectroscopy methods also show that the InAs/GaAs_1−_*_x_*Bi*_x_* heterosystem has a large lattice relaxation compared to that of InAs/GaAs. It is demonstrated that the surface diffusion of In increases during ion-beam deposition of InAs QDs on the GaAs_1−_*_x_*Bi*_x_* surface.
